# Endoscopic-assisted combined transcrusal anterior petrosal approach for resection of large petroclival meningioma: operative video and nuances of technique

**DOI:** 10.3171/2022.1.FOCVID21257

**Published:** 2022-04-01

**Authors:** Ali Tayebi Meybodi, James K. Liu

**Affiliations:** Department of Neurological Surgery, Center for Skull Base and Pituitary Surgery, Rutgers New Jersey Medical School, Newark; and; Saint Barnabas Medical Center, RWJ Barnabas Health, Livingston, New Jersey

**Keywords:** petroclival meningioma, combined transcrusal anterior petrosal approach, skull base surgery, endoscopic-assisted, combined petrosal, Kawase’s approach

## Abstract

In this illustrative video, the authors demonstrate an endoscopic-assisted combined transcrusal anterior petrosal approach for resection of a large petroclival meningioma with significant brainstem compression involving Meckel’s cave. This unique petrosal variant provides increased petroclival exposure that can potentially preserve hearing by combining a transcrusal labyrinthectomy with anterior petrosectomy (Kawase’s approach). The advantages include multidirectional angles of attack to the brainstem and petroclival region without cerebellar retraction. Endoscopic assistance allows expanded visualization into deep surgical corridors. The surgery was performed in a two-stage fashion, and a near-total resection was achieved with cranial nerve and hearing preservation. The operative nuances are demonstrated.

The video can be found here: https://stream.cadmore.media/r10.3171/2022.1.FOCVID21257

## Transcript

This is Dr. James Liu, and I will be demonstrating the endoscopic-assisted combined transcrusal anterior petrosal approach for resection of a large petroclival meningioma.

### 0:32 Patient History and Neurological Examination.

The patient is a 58-year-old female, who presented with headaches, significant gait ataxia, and multiple falls. Her neurological exam was otherwise nonfocal.

### 0:42 Preoperative Imaging.

MRI scan demonstrated a large left-sided petroclival meningioma with significant brainstem compression and obstructive hydrocephalus from effacement of the fourth ventricle. There is also tumor invading Meckel’s cave.

### 0:55 Choice of Surgical Approaches and Approach Selection Rationale.

Surgical approaches for this tumor include the retrosigmoid approach with intradural suprameatal drilling to Meckel’s cave. However, this approach has limited access to the medial brainstem region without significant brain retraction. Alternatively, one can consider petrosal approaches including the variations of the posterior petrosal approach and the anterior petrosal (Kawase’s) approach.^1–9^ We chose a combined petrosal approach combining the transcrusal and Kawase’s petrosectomy to give maximal petrous bone resection while potentially preserving hearing and minimizing cerebellar retraction. The transcrusal approach involves removal of the superior and posterior semicircular canals.^5,7^ This approach combined with the anterior petrosectomy provides wide multidirectional angles of attack to the brainstem and petroclival region, with a shorter distance to the petrous apex and Meckel’s cave. The use of endoscopic assistance also allows expanded visualization in and around deep corridors. Our operation was performed in a two-staged fashion.

### 2:03 Position and Skin Incision.

The patient is placed in the supine position with the head turned to the opposite side approximately 60° with the head in three-pin fixation. A retroauricular C-shaped incision, as well as abdominal fat and fascia lata incisions, are marked. Image guidance and neurophysiological monitoring are used.

### 2:22 Soft-Tissue Exposure and Combined Petrosal Approach.

A galeocutaneous flap is elevated and the temporalis muscle and fascia are reflected using a Z-shaped incision to expose the mastoid and squamosal portions of the temporal bone. A temporal craniotomy is performed, followed by an extended mastoidectomy that skeletonizes the sigmoid sinus and exposes both pre- and retrosigmoid dura. This is important to allow posterior mobilization of the sigmoid sinus to widen the presigmoid corridor.

### 2:52 Anterior Petrosectomy (Kawase’s Approach).

The temporal lobe dura is carefully elevated from the middle fossa floor, and the middle meningeal artery is coagulated and divided at the foramen spinosum. Extradural elevation from a posterior to anterior direction reveals the greater superficial petrosal nerve, that can be confirmed with the facial nerve stimulator at the geniculate ganglion. Once the middle fossa rhomboid is identified, we can begin the anterior petrosectomy with a high-speed diamond drill and copious irrigation. It is important to avoid drilling into the basal turn of the cochlea and the horizontal petrous ICA.

### 3:30 Transcrusal Petrosectomy.

At this juncture, we have completed an anterior and retrolabyrinthine posterior petrosectomy.^1,2^ A transcrusal petrosectomy is performed by removing the superior and posterior semicircular canals.^5,7^ The exposed ends of each canal are flooded with irrigation and filled with bone wax. It is thought that detailed attention to the prevention of an endolymph leak is important to hearing preservation using this technique. Drilling is continued to the common crus and the ampullated ends of both canals. Care is taken to avoid entering the lateral semicircular canal and the vestibule. This completes the combined transcrusal anterior petrosectomy with stable ABRs at baseline. Standard closure is performed, and the patient is left intubated and brought back to the OR the following day for the second stage of the procedure.

### 4:30 Petrosal Dural Opening With Tentorial Division.

A presigmoid and low temporal dural opening is performed. Care is taken to avoid injury to the vein or veins of Labbé. Here, the vein is adherent to the dura, so a relaxing cut is made to allow mobilization of the vein toward the temporal lobe to avoid injury. The superior petrosal sinus is ligated with a suture ligature and subsequently divided. The cut is continued along the tentorium toward the free edge of the incisura.

### 5:05 Tumor Removal.

The arachnoid is opened sharply to reveal cranial nerves VII to XI. We begin tumor dissection and debulking with an ultrasonic aspirator, working above the seventh nerve. As we continue dissecting the tumor away from the brainstem surface, we encounter the root of the trigeminal nerve that is displaced inferiorly by the tumor. Dissection is carried along the course of the cisternal segment of the trigeminal nerve. More of the brainstem is decompressed and we eventually expose the basilar artery. The fibrous ring of the porous trigeminus is opened sharply to expose the tumor invading into Meckel’s cave. The expansion of this corridor allows easy access to the tumor situated medial to the Gasserian ganglion within Meckel’s cave. The superior and medial pole of the tumor is exposed in the subtemporal corridor. A microscissors is used to incise the arachnoid and bring the tumor down into the field. We encounter the trochlear nerve, which has been displaced medially and superiorly. Sharp dissection of the arachnoid adhesions along the course of the nerve is critical to free up and separate the nerve from the tumor. We continue working on the anteromedial side of the tumor, peeling it away from the brainstem. In the petroclival region, we eventually identify the abducens nerve. Once the tumor is dissected free from the abducens nerve, the remainder of the tumor is dissected away from the petrous apex and Meckel’s cave. The remainder of the superior pole of the tumor is brought down by lysing the arachnoid adhesions. Care is taken to preserve the superior cerebellar artery. The last portion of tumor is carefully dissected away from the brainstem using gentle bipolar spreading technique, and single open-blade microscissors arachnoid dissection technique is a very efficient way of freeing up the tumor from neurovascular adhesions. We achieved a near-total resection, leaving tiny microscopic residue that was adherent to the trigeminal rootlets in Meckel’s cave. A 30° endoscope is used for final inspection of the resection cavity and confirms preservation of all the cranial nerves. We have a great view into Meckel’s cave anteriorly, with also confirmation of the trochlear nerve subtemporally.

### 8:39 Skull Base Reconstruction and Closure.

The mastoid antrum and air cells are waxed off, and the presigmoid dural defect is repaired using a fascial sling technique.^10^ A fascia lata graft is sutured into the dural defect to create a sling to hold the fat graft in place. Strips of fat graft are meticulously layered to prevent CSF leakage and to obliterate the mastoidectomy dead space. A Medpor Titan cranioplasty is used to reconstruct the bony defect and to bolster the fat graft in place.

### 9:17 Postoperative Course and Imaging.

Postoperatively, the patient was awake, alert, moving all extremities, with mild left facial weakness and decreased hearing on the left. Immediate postoperative MRI showed excellent decompression of the brainstem without any gross evidence of residual tumor. At the 3-month follow-up, she had significant improvement in gait, with normal facial nerve function. Formal audiogram demonstrated speech reception threshold of 60 dB and 84% word discrimination score. Sound booth testing showed improvement to 98% speech recognition score with a hearing aid. MRI scan at 3 months showed no gross evidence of residual or recurrent disease, which remained stable at 3 years postoperatively.

### 10:04 Conclusions.

In summary, the combined transcrusal anterior petrosal approach with endoscopic assistance is an effective strategy for treating complex tumors of the petroclival region.

## Disclosures

The authors report no conflict of interest concerning the materials or methods used in this study or the findings specified in this publication.

## Author Contributions

Primary surgeon: Liu. Editing and drafting the video and abstract: both authors. Critically revising the work: both authors. Reviewed submitted version of the work: both authors. Approved the final version of the work on behalf of both authors: Liu. Supervision: Liu.
